# Sensory Processing Across Conscious and Nonconscious Brain States: From Single Neurons to Distributed Networks for Inferential Representation

**DOI:** 10.3389/fnsys.2018.00049

**Published:** 2018-10-11

**Authors:** Umberto Olcese, Matthijs N. Oude Lohuis, Cyriel M. A. Pennartz

**Affiliations:** ^1^Cognitive and Systems Neuroscience Group, Swammerdam Institute for Life Sciences, Faculty of Science, University of Amsterdam, Amsterdam, Netherlands; ^2^Research Priority Area Brain and Cognition, University of Amsterdam, Amsterdam, Netherlands

**Keywords:** brain states, consciousness, neural representations, functional connectivity, ensemble activity, electrophysiology, perception, inference

## Abstract

Neuronal activity is markedly different across brain states: it varies from desynchronized activity during wakefulness to the synchronous alternation between active and silent states characteristic of deep sleep. Surprisingly, limited attention has been paid to investigating how brain states affect sensory processing. While it was long assumed that the brain was mostly disconnected from external stimuli during sleep, an increasing number of studies indicates that sensory stimuli continue to be processed across all brain states—albeit differently. In this review article, we first discuss what constitutes a brain state. We argue that—next to global, behavioral states such as wakefulness and sleep—there is a concomitant need to distinguish bouts of oscillatory dynamics with specific global/local activity patterns and lasting for a few hundreds of milliseconds, as these can lead to the same sensory stimulus being either perceived or not. We define these short-lasting bouts as micro-states. We proceed to characterize how sensory-evoked neural responses vary between conscious and nonconscious states. We focus on two complementary aspects: neuronal ensembles and inter-areal communication. First, we review which features of ensemble activity are conducive to perception, and how these features vary across brain states. Properties such as heterogeneity, sparsity and synchronicity in neuronal ensembles will especially be considered as essential correlates of conscious processing. Second, we discuss how inter-areal communication varies across brain states and how this may affect brain operations and sensory processing. Finally, we discuss predictive coding (PC) and the concept of multi-level representations as a key framework for understanding conscious sensory processing. In this framework the brain implements conscious representations as inferences about world states across multiple representational levels. In this representational hierarchy, low-level inference may be carried out nonconsciously, whereas high levels integrate across different sensory modalities and larger spatial scales, correlating with conscious processing. This inferential framework is used to interpret several cellular and population-level findings in the context of brain states, and we briefly compare its implications to two other theories of consciousness. In conclusion, this review article, provides foundations to guide future studies aiming to uncover the mechanisms of sensory processing and perception across brain states.

## Introduction

Our senses are always active. Even when we are deeply asleep, a salient stimulus such as an alarm clock is able to elicit a behavioral response. Weak stimuli, on the other hand, are not noticed, although they would normally be perceived during wakefulness. Also during deep surgical anesthesia, stimuli are still processed by multiple neocortical areas (Alkire et al., 2008; Mohajerani et al., 2011; Supp et al., 2011; Koch et al., 2016), yet they are not consciously perceived (Sanders et al., 2012). The awake state is similarly puzzling. We are able to process even the fine details of a visual scene easily, but sometimes we surprisingly fail to detect highly salient objects (Simons and Chabris, 1999).

What are the mechanisms underlying such a high variability in the way the same sensory stimulus is processed, and how are they relevant for understanding perception? A key factor to consider is the interaction between brain state and signals originating from sensory transducers. While the latter have been extensively investigated, the nature of brain states and how they influence sensory processing has received surprisingly limited attention. Historically, most studies on sensory processing in human subjects and primates have been performed in the awake state. Conversely, the majority of studies performed in other mammals (e.g., cats, rodents, ferrets) were done under anesthesia. Only in the last decade have researchers started to perform experiments in head-fixed rodents—a pre-requisite for the controlled delivery of sensory stimuli—not only during anesthesia, but also in awake, behaving animals (Carandini and Churchland, 2013; Guo et al., 2014; Montijn et al., 2015). Recent studies revealed that sensory-evoked responses are different between wakefulness and anesthesia (Alkire et al., 2008; Harris and Thiele, 2011), but also call for an update of the very definition of brain state. Neither wakefulness nor the various sleep stages (NREM stages 1, 2, 3 and REM) can be considered homogeneous brain states, as each of them is characterized by the co-existence of different neural dynamics both in time and across brain regions (Vyazovskiy et al., 2009, 2011; Nobili et al., 2012; Hung et al., 2013; Vyazovskiy and Harris, 2013).

The aim of this review article is threefold. First, we will develop an updated definition of brain state. We will argue that the fine-grained structure of brain activity (both in temporal and spatial terms) as a whole is what determines how a sensory stimulus is processed. As this activity structure is ever-changing, a single micro-state may last for only a few hundreds of milliseconds and should be ultimately functionally defined, based not only on the specific pattern of spontaneous activity, but also on how a given signal gets processed. Second, we will review how sensory processing is shaped by the characteristics of brain states, in terms of single-neuron and population responses, as well as communication between areas. Overall, we will present an overview of how stimuli are processed in single (cortical) sensory areas, and how sensory information propagates and reverberates across the cortical hierarchy to be consciously perceived and/or elicit a behavioral response. Finally, we will discuss what enables certain brain states to transform sensory information into conscious experiences, and how this is reflected in terms of neural representation of sensory stimuli.

With “consciousness” we indicate a state in which we experience the world—including our body—in a qualitatively rich manner (Pennartz, 2018). Several forms of experience, and corresponding states, are distinguished: (i) perception; (ii) imagery; and (iii) dreaming. Whereas perception is understood to be driven by external stimuli, imagery and dreaming are internally driven classes of experience. These manifestations of consciousness contrast with nonconscious states such as dreamless sleep, anesthesia or coma. Because the current article focuses on sensory processing, the ensuing discussion will mainly revolve around mechanisms of perception. We follow Jackendoff (1987) in that conscious experience (including dreaming and imagery) is essentially defined by its sensory nature. However, sensory processing is not sufficient for consciousness to arise, because it can also occur non-consciously. An important distinction is that between the state of consciousness (e.g., dreaming, NREM sleep) and its contents (what we are conscious of). When attempting to define consciousness or experience, one invariably risks to resort to circularity.

To avoid falling into this trap, we have previously (Pennartz, 2015) characterized experience as having a number of properties, at least in a state of healthy, full-blown consciousness, which we may summarize as follows: (i) experience is qualitatively rich in the sense that contents are set in multiple sensory modalities (visual, auditory, tactile, olfactory, gustatory, vestibular) and submodalities (e.g., color and shape within vision); with this richness come the properties of immersiveness and situatedness (the experience that the subject is immersed in a particular spatiotemporal situation—the basic realization of being somewhere at a particular time); (ii) what we experience depends on the interpretation of sensory input in terms of objects and events unequal to the neural substrate itself; this means that experiences are about something else than the neurons coding these very experiences (intentionality; Searle, 1983, 2004); and (iii) experience is integrative (Tononi, 2004) and set in a first-person perspective, i.e., the various elements consciously experienced are unified within an overall percept that is experienced from a common subjective sensing position (e.g., a viewpoint integrated with body position in space; Pennartz, 2015, 2018). There have been many proposals to ground consciousness not only on brain processes, but also in behavior and the external environment (e.g., O’Regan and Noë, 2001); because this review article deals with sensory processing in neural systems, we focus on theories of consciousness which attempt to explain which properties of neural systems may give rise to conscious processing (Pennartz, 2018). In particular, we will highlight inferential accounts of consciousness (Hobson and Friston, 2014; Pennartz, 2015) while also referring to Global Neuronal Workspace (GNW) theory (Dehaene and Changeux, 2011) and Integrated Information theory (IIT; Tononi et al., 2016).

Experiments in head-fixed rodents have recently enabled researchers to uncover several microcircuit-level principles underpinning sensory processing, to a degree of characterization that is not achievable in other mammals—thanks to techniques such as ensemble recordings, two-photon imaging and optogenetics (Denk et al., 1990; Wilson and McNaughton, 1993; Bernstein and Boyden, 2011; Vinck et al., 2015b). For this reason, we will focus on *in vivo* studies on rodent cortical physiology, and extend these results to the human case. Also, we will primarily focus on studies of sensory processing in the thalamocortical system, as this is considered to be the key brain system to generate conscious percepts (Koch et al., 2016). Previously, we have also argued why rodents offer a suitable model to study sensory processing in relation to consciousness (Storm et al., 2017). In brief, the typical electrophysiological and behavioral markers of the presence/absence of consciousness are present in both rodents and humans (Seth et al., 2005; Storm et al., 2017), and there is a strong anatomical homology between rodent and human brains. All of this suggests that rodents possess the key requirements to sustain conscious processing, and thus enable to investigate the underlying neuronal mechanisms.

## What Defines a Brain State?

**Figure 1:**
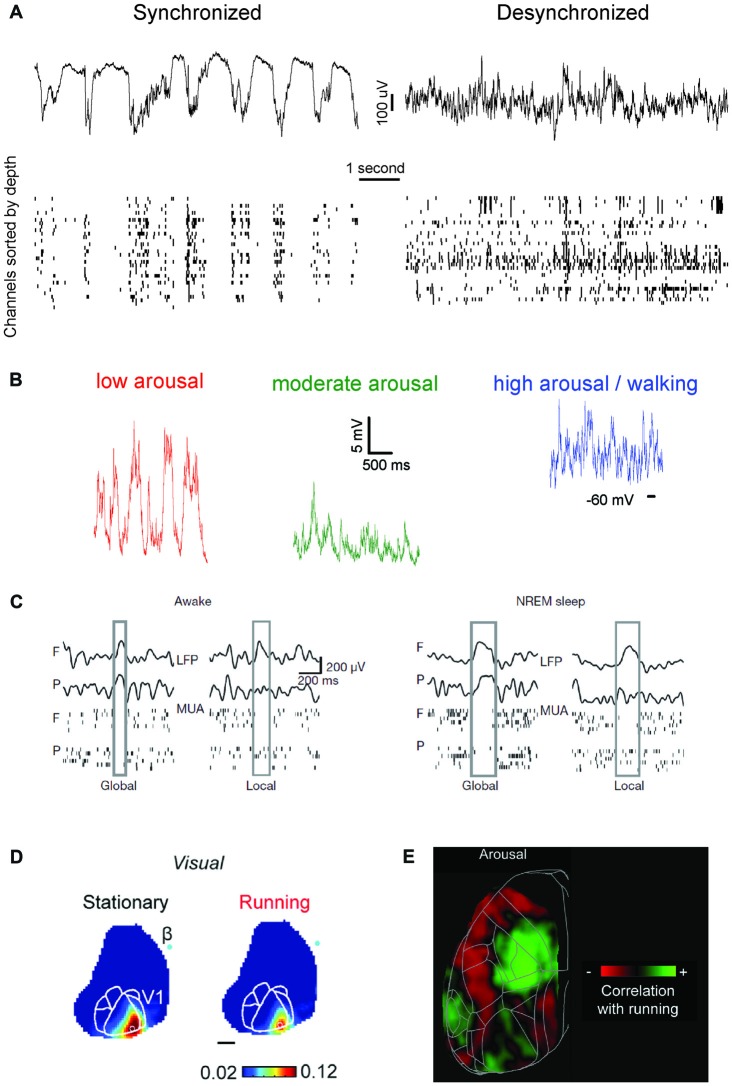
An updated definition of brain states. **(A)** Brain states have traditionally been distinguished based on the characteristic of population-level oscillatory dynamics present within a behaviorally-defined homogeneous period. Left, top: local field potential (LFP) trace present in mouse visual cortex during isoflurane anesthesia. LFP activity is characterized by oscillatory dynamics with a strong power in slow frequencies (0.5–4 Hz). Left, bottom: at the neuronal level, isoflurane anesthesia determines the alternation of periods of spiking and silence which are synchronous throughout cortical areas, and in phase with the co-occurring LFP oscillations. Right: same as left panel, for activity typical of wakefulness. Note the disappearance of slow frequency oscillations at the LFP level and the overall loss of synchrony for neuronal activity. **(B)** Recent studies have shown that, within wakefulness, much more variability is present than previously thought. In periods characterized by a low arousal level (left) neuronal activity (here shown in terms of intracellular membrane potential traces) displays patterns indistinguishable from those present during Non-REM sleep or anesthesia. As the arousal level increases, or if a period of activity—locomotion—occurs (center, right) activity becomes more and more desynchronized. Wakefulness can thus be subdivided in several micro-states, with markedly different properties. Adapted from McGinley et al. (2015b), permission to reproduce has been obtained from the publisher. **(C)** During Non-REM sleep and forms of non-dissociative (e.g., isoflurane) anesthesia UP and DOWN states normally occurs globally, i.e., involving the whole thalamocortical system. However, UP/DOWN states can also be local events, involving one a set of cortical regions. This mostly occurs during wakefulness when homeostatic sleep pressure is high (left), and during Non-REM sleep when, conversely, sleep pressure is slow. Each plot shows an example of global or local DOWN state occurring during wakefulness (left) or sleep (right), and measured in terms of both LFP recordings or neuronal multi-unit activity (MUA). F: frontal derivation. P: parietal derivation. Adapted with permission from the authors from Vyazovskiy et al. (2011). **(D)** Sensory processing within wakefulness varies across distinct micro-states. Visual evoked potentials (here shown as measure by voltage-sensitive fluorescent proteins) are generally weaker when a mouse is running compared to when it is stationary. The same is observed for audition and somatosensation. B: Bregma. Adapted under Creative Commons Attributions CC BY 4.0 license from Shimaoka et al. (2018). **(E)** Arousal differentially affects cortical areas in the mouse. Green indicates cortical areas where the signal measured via voltage-sensitive fluorescent protein is positively correlated with arousal (locomotion). Red shows areas where a negative correlation is present. Adapted under Creative Commons Attributions CC BY 4.0 license from Shimaoka et al. (2018).

Traditionally, brain states have either been associated with behavioral states, or with a predominant form of brain dynamics, often defined in terms of cortical activity (Pace-Schott and Hobson, 2002; Sabri and Arabzadeh, 2018). Importantly, these two characterizations of brain state often correlate. Wakefulness is classically characterized by the presence of desynchronized neural activity (McGinley et al., 2015b), see Figure 1A. At the single-neuron level, this desynchronization is manifested as tonic spiking and prolonged depolarizations (Steriade et al., 2001). NREM sleep (as well as forms of non-dissociative anesthesia) is instead characterized by massive neural synchrony, which progressively increases from light to deep sleep and becomes prominent during stage N3 (Berry et al., 2017)—also referred to as slow wave sleep (SWS). During SWS, cortical activity is dominated by synchronous oscillations below 4 Hz (delta and slow wave ranges, Crunelli and Hughes, 2010; Olcese and Faraguna, 2015) and, at the single-neuron level, by the alternation of hyperpolarized DOWN states and depolarized UP states (Steriade et al., 2001), see Figure 1A. In contrast, REM sleep is characterized—similarly to wakefulness—by desynchronized activity, and by specific neurophysiological features which include theta oscillations in the hippocampus, ponto-geniculo-occipital (PGO) waves, muscle atonia and rapid eye movements (Pace-Schott and Hobson, 2002). In the last decade, this picture of brain states has been upended, as an increasing number of studies has shown that brain states are not monolithic, homogeneous entities, as was previously thought.

### The Ever-Changing Nature of Neuronal Activity Patterns

Brain states are highly dynamic. While this is obvious for the waking state—which we will extensively cover in the next subsection—all brain states show variable forms of neural dynamics. Here we will focus on SWS, the deepest stage of NREM sleep, which is largely associated with a loss of consciousness—although dreaming can also occur during this state (Hobson et al., 2000; Siclari et al., 2012, 2017). During slow oscillations, the cortical hallmark of SWS (Steriade et al., 2001; Compte et al., 2003), neurons synchronously undergo a periodic shift between a hyperpolarized and a depolarized state. Sensory stimuli occurring during either an UP or DOWN state will inevitable face a distinct fate. Therefore, rather than taking NREM sleep as a unitary brain state, up and down states should themselves be considered as individual micro-states, embedded within a behavioral state. Furthermore, UP and DOWN states have a variable duration, ranging from tens to hundreds of milliseconds, and show a varying degree of synchrony (Esser et al., 2007; Vyazovskiy et al., 2009). These properties are not randomly distributed throughout SWS, but conform to the time course of sleep homeostasis (Vyazovskiy et al., 2009)—or “process S” (Borbély, 1982). This can be unpacked as follows. During sleep early in the night, when sleep pressure is high, up states are shorter and more synchronous than during late sleep (Vyazovskiy et al., 2009). A computational study suggested that this process is indicative of higher synaptic strength (Olcese et al., 2010), with consequences on neural computations. Moreover, reactivation of memory traces during NREM sleep, which has been indicated as a mechanism underlying sleep-dependent memory consolidation, primarily occurs in early sleep (Kudrimoti et al., 1999; Ji and Wilson, 2007; Lansink et al., 2008, 2009). Thus, not only up and down states can be considered to constitute different “micro” brain states but a further distinction might be made based on when these occur along the wake-sleep cycle.

UP and DOWN states are not the only form of sleep-related neural oscillation displaying a local nature. Sleep spindles, a hallmark of NREM stage 2 in humans, have also been shown to occur locally (Andrillon et al., 2011; Nir et al., 2011). Sleep spindles play a role in modulating thalamic gating and tolerance to external stimuli (i.e., the fact that subjects are not awakened by them; Dang-Vu et al., 2010a). Similar to slow oscillations, sleep spindles therefore play an essential role in modulating cortical responsiveness to external stimuli, and may be considered a separate “micro” state within NREM stage 2.

While UP and DOWN states (and also spindles) are typically associated with thalamocortical oscillations (Steriade, 2003; Crunelli and Hughes, 2010; Sanchez-Vives et al., 2017), distinct forms of neural activity are present in other brain regions. One prime example are sharp-wave ripples, high-frequency (about 150 Hz) hippocampal oscillations which have been implicated in memory consolidation (Buzsáki, 2015). While sharp wave ripples are temporally locked to cortical slow oscillations (Mölle et al., 2006; Clemens et al., 2007; Mölle and Born, 2011), they do not co-occur with every UP/DOWN state. Therefore, slow oscillations which either co-occur or not with a sharp wave ripple might be considered distinct micro-states.

Along the same line, transition periods from one behavioral state to another show intermediate features of both states (Lewis et al., 2012; Marzano et al., 2013; Bettinardi et al., 2015; Emrick et al., 2016; Stitt et al., 2017). For example, the cortex can show NREM-like activity while the hippocampus does not (Emrick et al., 2016). Strikingly, even within the neocortex, some areas can display local, wake-like behavior when the rest of the cortex shows slow-wave activity and subjects are behaviorally asleep (Nobili et al., 2012), or vice versa (Vyazovskiy et al., 2011), see Figure 1C. Thus, each unique spatiotemporal pattern of activity occurring in a brain area may ultimately determine how information is processed by the rest of the brain and needs to be taken into account to devise a definition of brain state.

This overview shows that brain activity during NREM sleep is highly heterogeneous, and that a myriad of distinct micro-states can be identified, based on their distinct spatiotemporal patterns of activity. This, however, would lead to an almost endless parcellation of behavioral, macro-states into these new micro-states. It is therefore apparent that neural patterns of activity are not—*per se—*sufficient to discriminate what constitute a “micro” brain state. A functional definition, based on whether distinct patterns of activity have consequences for how the brain operates, and what it computationally achieves, appears more fruitful. To explore this, we will now discuss whether wakefulness, the state in which such functions can be more easily probed, also shows a subdivision into putative micro-states.

### The Strange Nature of Wakefulness

An apparently homogeneous behavioral state such as SWS appears to rather be a collection of several diverse micro-states. The same observation applies to wakefulness. Indeed, wakefulness is hardly definable as a single behavioral state. When we are awake, we can either sit quietly and mind-wander or be highly involved in a myriad of different activities, from running to thinking. A key factor which varies during wakefulness is the arousal level (Reimer et al., 2014; McGinley et al., 2015a,b), as a function of which different levels of cholinergic and noradrenergic activity (amongst other neuromodulators) modulate baseline neuronal activity and consequently the way in which sensory stimuli are processed.

The arousal level is primarily measured in terms of pupil diameter, with a larger pupil being associated with higher arousal (Reimer et al., 2014; McGinley et al., 2015b). High arousal is accompanied by desynchronized cortical activity, while during low arousal neural patterns become more synchronized (McGinley et al., 2015b), to the point that slow oscillations (the hallmark of NREM sleep) occur during quiet wakefulness (Petersen et al., 2003; Sachidhanandam et al., 2013; McGinley et al., 2015a), see Figure 1B. Locomotion usually corresponds to a state of high arousal (measured in terms of pupil size), although the two are not unequivocally linked, at least as far as visual processing is concerned (Vinck et al., 2015a). During locomotion, visual responses are, similar to periods with large pupil diameter, enhanced (Niell and Stryker, 2010; Dadarlat and Stryker, 2017; Kaneko et al., 2017), yet via a distinct mechanism. While pupil-related arousal suppresses spontaneous firing activity (thus promoting the emergence of stimulus-evoked responses), locomotion increases stimulus-evoked activity (Bennett et al., 2013; Vinck et al., 2015a; Dadarlat and Stryker, 2017). Although differences exist in the way brain state modulates sensory processing in each modality across distinct cortical areas (Shimaoka et al., 2018)—see Figures 1D,E—both locomotion and arousal cause an overall enhancement of sensory processing. Moreover, the presence of stimuli (e.g., visual ones) affects the way in which locomotion modulates neuronal activity (Pakan et al., 2016), suggesting that sensory context modulates the effect of brain state, in a recursive, complex manner.

Differences between arousal levels are not limited to cortical activity. Indeed, hippocampal sharp wave ripples are almost absent during active wakefulness, but strongly present during quiet awake periods (Buzsáki, 2015; Roumis and Frank, 2015)—although they might play a different role in wakefulness than in NREM (Roumis and Frank, 2015). Intriguingly, the transition that neuronal activity patterns undergo between low and high arousal states is not clearly understood, as several contrasting models (binary, sigmoid and U-shaped) have been proposed (McGinley et al., 2015b). An intermediate level of arousal has been associated with optimal sensory processing (Yerkes and Dodson, 1908; McGinley et al., 2015a), suggesting that neither highly synchronous nor fully desynchronized activity is optimal.

Related to arousal, yet distinct from it, is the level of attention paid during a sensory processing task (Harris and Thiele, 2011). The term “engagement” is often used in the rodent literature when comparing sensory evoked activity between animals trained to pay attention to sensory stimuli and naïve ones (Sachidhanandam et al., 2013; Carcea et al., 2017; Kuchibhotla et al., 2017).

Top-down attention has been extensively investigated (Buschman and Miller, 2007; Buschman and Kastner, 2015), and is known to locally modulate brain state by promoting desynchronized activity (Harris and Thiele, 2011; Ecker et al., 2016). It has been hypothesized that three attentional states exist: (1) a state of low attention, during which neural activity is highly synchronous and slow oscillatory patterns appears (Harris and Thiele, 2011); (2) a rhythmic mode of attention (Schroeder and Lakatos, 2009)—also referred to as transient or scanning attention (Aston-Jones et al., 1999, 2000)—characterized by oscillatory patterns entrained to task-relevant sensory stimuli (usually in the theta-band range, Schroeder and Lakatos, 2009); (3) a continuous/sustained mode of attention (Aston-Jones et al., 1999, 2000; Schroeder and Lakatos, 2009; Fries, 2015), characterized by desynchronized activity. The transitions between these different attentional states, similarly to transitions between sleep and wakefulness (Pace-Schott and Hobson, 2002), are, at least in part, regulated by noradrenergic influences originating in the locus coeruleus (Aston-Jones et al., 1999, 2000; Harris and Thiele, 2011), and suggest therefore that all these states are part of a continuum: from fully synchronized activity during SWS to fully desynchronized patterns during sustained attention.

### An Updated Definition of Brain States

In the previous sections we showed how neural activity during both NREM sleep and wakefulness displays a wide variety of patterns. Here, for the sake of brevity, we focus on the two most prominent conscious and nonconscious behavioral states (wakefulness and NREM sleep, respectively), but similar conclusions (e.g., about the local nature of brain states) can be drawn for REM sleep (Funk et al., 2016). Thus, “macroscopic” behavioral states (wakefulness, SWS, REM, etc.) are not homogeneous, unitary phases in brain activity, but can rather be further subdivided into distinct “micro”-states (see Table 1). But what defines such micro-states?

**Table 1:** Macro- and micro-states.

Macro-states	Wakefulness	REM sleep	Non-REM sleep			Anesthesia	
			N1	N2	N3/SWS	Non-dissociative (e.g isoflurane, urethane)	Dissociative (e.g., ketamine)
**Micro-states**	Low arousal	Local slow oscillations in neocortical circuits	Global UP/DOWN states and spindles			Global, rhythmic UP/DOWN states, spindles	Sporadic UP/DOWN states, gamma bouts
	Medium arousal High arousal/locomotion						
	Theta/alpha bouts		Local UP/DOWN states and spindles				

*This table presents an overview of the most relevant micro-states, i.e., subdivisions of the generally considered (macro-) brain states, based on the effect they have on how sensory information is processed. We thus here primarily refer to electrophysiological features of neocortical circuits. N1, N2 and N3 corresponds to the different stages of Non-REM sleep, based on Berry et al. (2017); N3, the deepest sleep stage, is also referred to as slow wave sleep (SWS); in rodents, Non-REM is usually not subdivided into stages*.

For example, neural dynamics during NREM UP states are so similar to those present in the awake desynchronized state that some authors have proposed that up states might represent “fragments” of wakefulness (Destexhe et al., 2007). During UP states, according to this view, sensory stimuli would be processed similarly to what happens during wakefulness, yet without being able to propagate across cortical areas, as a consequence of the breakdown in cortical communication that has been suggested to occur during NREM and nonconscious states (Massimini et al., 2005; Casarotto et al., 2016). If a brain state were to be defined only in terms of activity patterns, UP states during NREM sleep and desynchronized activity in wakefulness could be classified as the same state.

Nevertheless, this position is debatable, as: (1) the neuromodulatory milieu is markedly different between wakefulness, NREM and REM sleep (Pace-Schott and Hobson, 2002); and (2) slow oscillatory dynamics occurring during quiet wakefulness show properties distinct from those found in NREM sleep (Vyazovskiy et al., 2011; Sachidhanandam et al., 2013; McGinley et al., 2015b). As a consequence, a sensory stimulus occurring during NREM sleep is able to recruit a slow oscillation (Riedner et al., 2011) and is not generally processed beyond the sensory cortices (Dang-Vu et al., 2010b), while the same stimulus during low-arousal (synchronous) wakefulness triggers a transition to a more desynchronized type of activity (Tan et al., 2014).

Thus, we favor a definition of micro-state based on how a given sensory stimulus is processed. Micro-states are embedded into macro-states. They share with them a general level of behavioral responsiveness, but further refine them in terms of brain/sensory function. This corresponds for example to the presence or absence of dreaming during either NREM or REM, or, specifically pertaining to sensory processing, to whether a given stimulus or its constituent features (e.g., the various details which compose the sensory scene) are perceived. A single micro-state can then be defined as a period of brain activity lasting at least a few hundreds of milliseconds—in line with the presumed duration of a conscious sensory experience (Tononi and Edelman, 1998)—and characterized by a uniform spatio-temporal pattern and neuromodulatory milieu, embedded into a sensory context, within which a given sensory stimulus is processed uniformly for as long as the state lasts. According to this definition, a single UP state during SWS (a macro-state) would qualify as a distinct micro-state, distinct from both DOWN states and from UP states occurring during quiet wakefulness. Similarly, different bouts of oscillatory dynamics critical for sensory processing (e.g., theta oscillations) will be considered individual brain states only if they markedly alter the way in which a sensory stimulus is processed, compared to different types oscillatory dynamics within a macro-state.

Importantly, the link between micro-states and sensory processing is bidirectional, as both can influence each other. For example, delivering sensory stimuli during quiet wakefulness triggers the transition to a depolarized, asynchronous state (Sachidhanandam et al., 2013; Tan et al., 2014), while K-complexes (Roth et al., 1956) and slow oscillations (Ngo et al., 2013, 2015) can be elicited by auditory stimuli during NREM sleep (but see, Goltstein et al., 2015). Thus, micro-states not only influence sensory processing, but sensory stimuli themselves can modify the state of brain networks and consequently affect how they are processed.

In the next sections, we will analyze how sensory stimuli are processed in different micro-state configurations. This will allow us to characterize how these subdivisions of behavioral macro-states affect sensory processing and perception.

## Sensory-Evoked Neuronal Responses Across Brain States

Sensory processing markedly changes across brain states. Here, we will focus on those changes that are most relevant with respect to conscious perception, and will review how they are related to variations in brain activity at various levels of circuital complexity (single neurons, ensembles, multi-area). We will first explore whether the breakdown of perception during nonconscious states (anesthesia or SWS) is due to the inability of single neurons to faithfully respond to sensory stimuli, recognizing the importance of considering variability between micro-states, such as UP or DOWN states. Although we can conclude that a more desynchronized state facilitates encoding of sensory features, also at the single neuron level, neurons still display responses tuned to specific sensory features throughout sensory cortices during nonconscious brain states. Thus, whether a single neuron fires across different states correlates very poorly to whether stimuli are perceived or not. Therefore, we subsequently consider what features of ensemble activity are conducive to forming conscious sensory representations and how activity propagates and reverberates across areas.

### Micro-State Affects Single-Neuron Dynamics

Ultimately, the brain’s ability to represent information about the outside world is based on its coding elements: neurons. We can thus ask the question of whether perception is lost during anesthesia or NREM states because sensory signaling is degraded, and therefore the system cannot compose accurate representations of the external world anymore. A host of studies has characterized sensory responses across synchronized and desynchronized states. Consistently, single neurons in primary sensory areas show remarkably similar tuning to stimulus features across behavioral states, e.g., neurons in visual cortex rarely shift their preferred orientation (Goard and Dan, 2009; Niell and Stryker, 2010; Ecker et al., 2014; Goltstein et al., 2015; Nir et al., 2015; Durand et al., 2016). In general, responses seem to have slightly faster dynamics in desynchronized brain states (i.e., shorter onset latency and faster transients; Wörgötter et al., 1998; Hasenstaub et al., 2007; Haider et al., 2013; Wang et al., 2014; Durand et al., 2016), but see (Pachitariu et al., 2015). Even though this suggests that major feedforward pathways are to a large extent functioning similarly throughout states, response dynamics can vary considerably when considering the immediate cortical micro-state that sensory inputs face when reaching the cortex.

Early work in the anesthetized cat visual cortex showed that identical stimuli can elicit different neuronal responses depending on whether they arrive during UP or DOWN states (Arieli et al., 1996; Azouz and Gray, 1999; Haider et al., 2007), but see (Haider et al., 2013). Also in the awake rodent somatosensory cortex responses to single whisker deflections are larger when occurring during DOWN states of synchronized activity, as opposed to UP states (Petersen et al., 2003; Sachdev et al., 2004; Crochet and Petersen, 2006; Hasenstaub et al., 2007; Sachidhanandam et al., 2013), similar to responses of neurons in auditory cortex to isolated tones (Deweese and Zador, 2004; Sela et al., 2016). Across repeated presentations of the same stimulus this leads to increased trial-to-trial variability, compared to the desynchronized state, during which fluctuations in population activity and network excitability are instead smaller (Haider et al., 2013; Zagha et al., 2013).

Also, within wakefulness the response of a single neuron to a stimulus dynamically varies between micro-states. As mentioned earlier, in mouse visual cortex active locomotion increases responsiveness to drifting gratings (Niell and Stryker, 2010; Bennett et al., 2013; Polack et al., 2013; Fu et al., 2014). In other sensory systems such as auditory cortex, locomotion results in significant suppression of sensory-evoked responses (Schneider et al., 2014; Zhou et al., 2014) and in somatosensory cortex active whisker movement reduces sensory responses to touching objects (Ferezou et al., 2007; Poulet and Petersen, 2008). These might represent ecologically relevant differences between sensory systems (see also Figure 1E), since the way cortical processing needs to accommodate changes in sensory input upon active behavior such as exploration or whisking may be modality-dependent.

Irrespective of sensory modality, heightened arousal seems to enhance the consistency and signal strength in the coding of stimuli (but see Shimaoka et al., 2018). Across identical stimulus presentations the variability in response is reduced when the arousal level is high (Polack et al., 2013; Schneider et al., 2014; Schölvinck et al., 2015; McGinley et al., 2015a)—see Figure 1D—with again a key contributor being reduced pre-stimulus variability (Bennett et al., 2013; Zagha et al., 2013). This, in combination with higher membrane conductance in the desynchronized state (Wang et al., 2014) or during locomotion (Bennett et al., 2013), can lead to an increased signal-to-noise ratio (response vs. baseline variance; Bennett et al., 2013; Pachitariu et al., 2015; Vinck et al., 2015a). These state-dependent alterations in single neuron coding correlate with increased performance in sensory detection tasks (Bennett et al., 2013; Pinto et al., 2013; McGinley et al., 2015a, but see Sachidhanandam et al., 2013).

**Figure 2:**
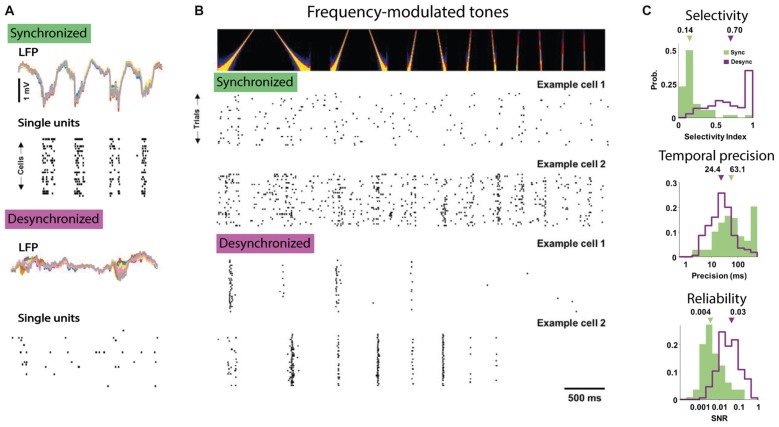
The desynchronized state facilitates feature coding by single neurons. **(A)** Example of simultaneous LFP and single neuron activity in anesthetized auditory cortex of gerbils in the synchronized (top) and desynchronized state (bottom). Note that in the synchronized state most single unit activity tracks the large-amplitude fluctuations in the LFP. **(B)** Responses to tones are more selective and reliable over stimulus repetitions in the desynchronized state. Top panel shows the auditory spectrogram of the frequency modulated tone and the bottom panel shows the responses in rasterplots for two example cells to repetitions of the same stimulus recorded in the synchronized (top) and desynchronized state (bottom). Note the temporally precise and selective activity of the two example cells in the desynchronized condition compared to the non-selective responses in the synchronized condition. **(C)** Three measures of improved feature coding by single neurons in the desynchronized state. Top responses to tones had higher direction selectivity index, e.g., preference for upward vs. downward frequency modulated sweeps (computed as: response to preferred direction − opposite)/(preferred + opposite). Middle: responses during the desynchronized state were more temporally precise in their spiking (computed as the amount of jitter necessary to render the response uninformative, low value is higher precision) and, bottom, had higher reliability (computed as the signal-to-noise ratio). All figures adapted under Creative Commons Attribution License from Pachitariu et al. (2015).

The resulting view is that the slow rhythmic fluctuations in network excitability and membrane potential during SWS, anesthesia or quiet wakefulness introduce large variability in sensory processing. Conversely, cortical desynchronization during active waking and arousal facilitates single neuron coding by reducing trial-to-trial variability and facilitating temporally precise and consistent signaling of sensory features. A recent study nicely illustrates this conclusion even though the authors compared synchronized and desynchronized epochs only within the anesthetized state (Pachitariu et al., 2015). In this study, single auditory cortical neurons carried much more information regarding which frequency-modulated tone was presented during desynchronized compared to synchronized states, as in the former responses were more selective to tone frequency and were more temporally precise and reliable across repetitions (Figure 2).

Most of the studies discussed above measured responses to isolated and salient stimuli. Interesting state-specific differences are revealed when varying stimulus parameters, such as intensity. For example, several studies suggest that during UP states responses to low-intensity sounds are boosted, while responses to high-intensity stimuli are attenuated (Issa and Wang, 2011; Reig et al., 2015; Sela et al., 2016; Meijer et al., 2017). This suggests that responses are normalized across intensity levels during UP states; during DOWN states, on the other hand, responses to weak stimuli are suppressed (Issa and Wang, 2011). Another state-specific difference arises when presenting repeated stimuli, instead of isolated stimuli. For example, repeated whisker stimulation at high, but not low, frequencies rapidly induced adaptation of sensory responses in barrel cortex of both anesthetized and quiescent rats (Castro-Alamancos, 2004). However, upon active whisking in awake animals or after stimulation of the brain stem reticular formation to induce a more desynchronized cortical state during anesthesia, sensory responses become smaller at low frequencies, but maintain their response amplitude at higher whisker stimulation frequencies (Castro-Alamancos, 2004). This means that only in the desynchronized state single neurons are able to faithfully signal stimuli that are part of a longer sequence. Preserving large initial responses to a single isolated stimulus in the synchronized cortical state (during quiet wakefulness or SWS) is beneficial, as it might allow the animal to respond while resting. The intricate details of ongoing sensory input, however, are at that moment of less relevance and need not be accurately processed (Harris and Thiele, 2011).

The overall conclusion is that the most marked differences in sensory processing between states arise when shifting from isolated stimuli to more naturalistic and complex stimuli. Stimuli of higher complexity are much more consistently encoded by single neurons in the desynchronized state, for instance randomly ordered whisker deflections in somatosensory cortex (Zagha et al., 2013), amplitude-modulated noise stimuli by the auditory system (Marguet and Harris, 2011) and natural scenes by visual cortex (Goard and Dan, 2009). It is therefore conceivable that simple stimuli are less instructive as to why perception is lost during some states as opposed to other. For example, a selective breakdown of top-down projections during nonconscious brain states would result in striking differences only when using temporally and spatially structured stimuli dependent on modulation by top-down inputs.

### Brain-State Modulation of Ensemble Activity

As we discussed the information-signaling capacities of single neurons increases during desynchronized activity. However, whether a single neuron responds to a stimulus—which still occurs during deep anesthesia—is not informative in explaining why some stimuli come to be perceived or not. As stimuli are likely coded by patterns of ensemble activity (Pouget et al., 2000; Pennartz, 2015), it is essential to understand at a population level how the representation of information varies across states. We will here discuss the impact of correlated variability between neurons and the precise temporal structure of ensemble activity for accurate perception.

#### High Noise Correlations During Synchronized Activity

Upon rhythmic oscillations in population firing, spiking activity is positively correlated between nearby neuronal pairs, i.e., neurons increase and decrease their firing together (Lampl et al., 1999). Oppositely, during a desynchronized state, neurons do not show this large synchronous modulation of firing rate and fire more or less independently (Renart et al., 2010). If these synchronous fluctuations persist in responses to stimuli, i.e., neurons correlate in how strongly they respond to repeated presentations of the same stimulus, these pairwise correlations are called noise correlations (Abbott and Dayan, 1999; Averbeck et al., 2006; Cohen and Kohn, 2011).

Noise correlations are generally high during anesthesia or otherwise synchronized states (Renart et al., 2010; Ecker et al., 2014), due to these common fluctuations in activity and primarily arise as a result of neurons transiently ceasing firing together (Mochol et al., 2015). However, noise correlations increase even under light anesthesia and in the absence of clear UP/DOWN states (Greenberg et al., 2008; Golshani et al., 2009; Goltstein et al., 2015), and also when focusing only on UP states (Renart et al., 2010). Noise correlations can also vary between different micro-states within wakefulness (Poulet and Petersen, 2008; Gentet et al., 2010). Specifically, noise correlations decrease upon locomotion (Erisken et al., 2014; Vinck et al., 2015a) or arousal induced by an air-puff (Vinck et al., 2015a). We will specifically focus on these differences, and we will refer to other studies for more details on how sensory processing is affected by noise correlations in the context of visual attention (Cohen and Maunsell, 2011; Harris and Thiele, 2011; Maunsell, 2015).

#### Desynchronization Improves Population Coding

Traditionally noise correlations have been argued to impair sensory processing (Zohary et al., 1994; Shadlen and Newsome, 1998), but recent theoretical work has shown that only certain noise correlations (termed differential correlations) are information limiting, namely those fluctuations that are identical to those generated by stimulus variations (Moreno-Bote et al., 2014; Kohn et al., 2016). Additionally, our work suggests that pairwise noise correlations are not inherently detrimental (Montijn et al., 2014), especially when considering them within the context of larger populations (Montijn et al., 2016a). This is because multidimensional noise correlations (covariability between neuronal triplets etc.) are more likely to be orthogonal to the dimension coding stimulus identity, limiting the effect on population readout in downstream areas (Montijn et al., 2016a).

**Figure 3:**
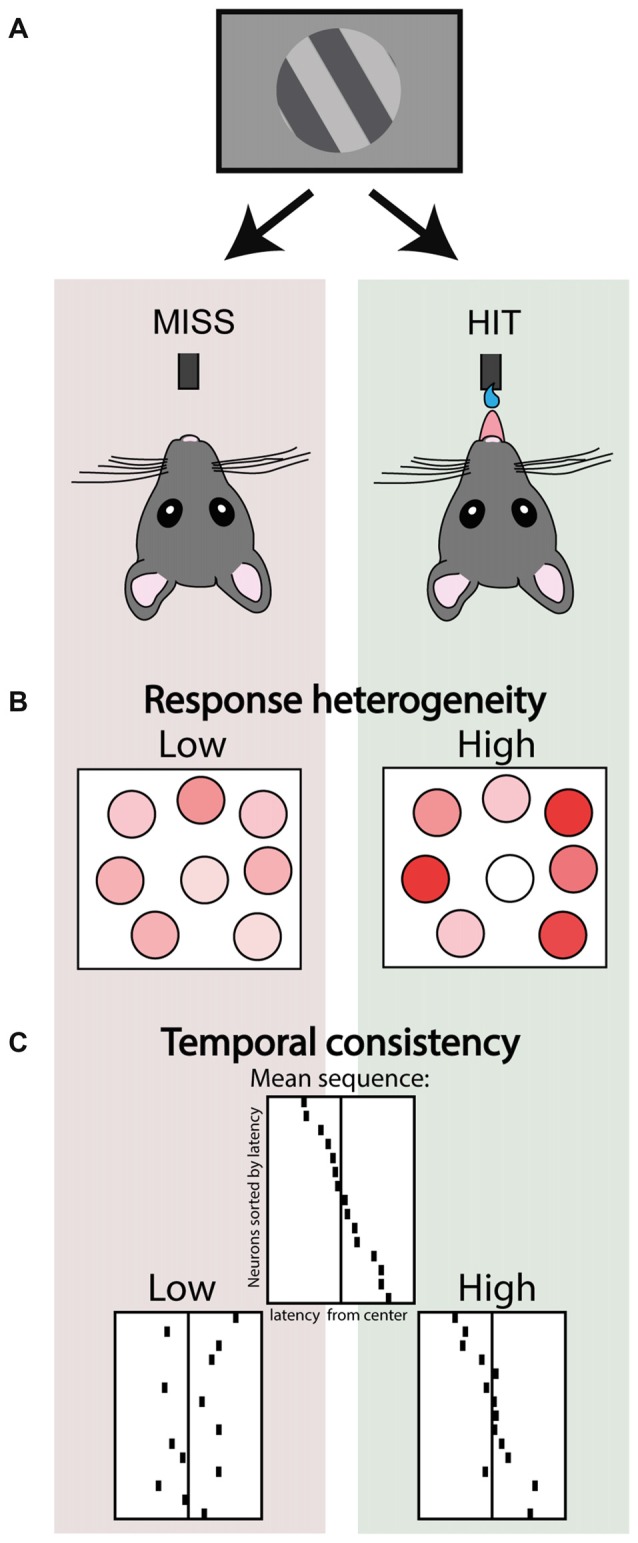
Ensemble activity correlates of stimulus detection. **(A)** Schematic depicting two trial outcomes in a visual detection task performed by head-fixed mice. In the left scenario a threshold stimulus is not detected (miss trial), whereas in the right the same stimulus is correctly detected (a rewarded hit trial). **(B)** Simultaneous 2-photon imaging of V1 neuronal populations in these mice revealed that hit trials were associated with higher response heterogeneity. Adapted under Creative Commons Attribution License from Montijn et al. (2015). **(C)** Structured ensemble activity was correlated with detection. Top center panel depicts a schematic of a neuronal ensemble showing a recurring stereotypical sequence of activity. Hit trials were associated with higher consistency and temporal precision of these recurring sequential events, compared to miss trials. Adapted with permission from the authors from Montijn et al. (2016b).

Nonetheless, the high noise correlations during synchronized activity do impair population coding. In macaque visual cortex a locally synchronized state just before stimulus onset impairs population readout of stimulus identity from a recorded ensemble as well as impairing behavioral performance (Beaman et al., 2017), and the amount of decorrelation upon visual input is correlated with detection of figure-ground stimuli (van der Togt et al., 2006). Experimentally promoting cortical desynchronization by nucleus basalis stimulation in rats improves population decoding of natural scenes (Goard and Dan, 2009), and performance in a visual discrimination task (Pinto et al., 2013). This suggests that any micro-state that facilitates independent coding of stimulus features and heterogeneous neuronal responses contributes to accurate perception. Indeed, the amount of relative contrast in activity between neurons, indexed as population response heterogeneity, correlates with hits vs. misses in a visual detection task (Montijn et al., 2015; Figure 3). This study showed that a highly heterogeneous activity pattern even preceded detected stimuli, whereas heterogeneity was lower during the pre-stimulus baseline of miss trials. This suggests that task epochs are characterized by different levels of heterogeneity in neural activity—they form different micro-states—and that these fluctuations might impact how incoming stimuli are subsequently processed.

While the improved population coding during desynchronized activity might contribute to accurate perception in sensory detection tasks, it is still present within non-conscious animals, such as when comparing desynchronized with synchronized epochs during urethane anesthesia (Goard and Dan, 2009; Pachitariu et al., 2015). Therefore, whether desynchronization *per se* contributes to perception remains to be addressed. Cortical state impacts the ability of a population of cells to extract sensory information, but whether this information is read out by downstream areas or generates a conscious representation probably depends on additional factors besides the overall synchronization level (for example, proper communication between brain areas), as we will discuss in “The Neuronal Basis of Conscious Sensory Processing” section.

#### Temporal Structure in Ensemble Activity

Neurons not only convey information by modulating their firing rate, but also via a precise timing of spiking activity (Hopfield, 1995; Montemurro et al., 2008; Kayser et al., 2009). Spike timing might be related to sensory stimuli, to mass neural dynamics (e.g., to the phase of the local field potential—LFP), or to the activity of other nearby neurons such that ensembles display stereotypical sequences of activation (first neuron A, then B, then C…). It has been argued that these “packets” of sequential neuronal activity lasting a few hundred milliseconds are actually the basis for information representation and communication in cortex (Luczak et al., 2015). Briefly, it can be noted that these activity packets are a local, spike-level manifestation of large-scale activity waves that travel across the cortex (Mohajerani et al., 2013). Packet-based communication has been proposed to be a more effective way of driving downstream target neurons (Luczak et al., 2015).

Interestingly, this highly structured sequential activity is found during both spontaneous and evoked activity, but also across brain states (Luczak et al., 2009). Likewise, we know from the phenomenon of memory replay that the same structured spiking sequences during a specific behavior can be replayed during subsequent resting and sleep periods (Skaggs and McNaughton, 1996; Pennartz et al., 2002; Ji and Wilson, 2007; Lansink et al., 2009). Neuronal populations thus maintain the capability of coordinated firing in highly structured temporal sequences during unconscious states.

The fact that stereotypical events re-occur in both conscious and unconscious states is partly explained by the fact that the realm of possible activity patterns is constrained by the anatomical connectivity of the microcircuit—which is arguably similar across states. But are these temporally-specific activity patterns also relevant to perception and do they correlate for example with behavior? In mice performing a visual detection task, we found that neuronal assemblies repeat specific sequential activity patterns (Montijn et al., 2016b). Rather than the recurrence of specific assemblies, the consistency of the temporal order in which they fired was associated with the animal perceiving a near-threshold stimulus or not (Montijn et al., 2016b; Figure 3). This suggests an important role for structured ensemble activity, but it is still relatively unexplored both: (1) what type of temporal structuring mechanisms are preserved and lost across behavioral states; and (2) how specific temporal ordering is ultimately associated with perception, for example by affecting downstream readout.

Before we explore how brain state affects communication between brain areas, we conclude that the desynchronized micro-state is marked by improved neural coding through both an increase in the trial-by-trial reliability of single neuron responses, and a decorrelation at the population level. Furthermore, within wakefulness decorrelated and heterogeneous neuronal activity patterns as well as consistent temporal sequences are associated with stimulus detection. The relevance of these phenomena for conscious sensory processing will be discussed in more detail in “The Neuronal Basis of Conscious Sensory Processing” section.

### How Communication Between Brain Areas Varies Across Brain States and How This Affects Sensory Processing

Although most research on sensory processing has been performed at the level of primary sensory cortices (as discussed above), these areas represent only a first step in the pathways for sensory cortical processing (Felleman and Van Essen, 1991; Markov et al., 2014; Oh et al., 2014; Glasser et al., 2016). After this stage, sensory information progresses to other areas, which also send back top-down information, and is ultimately transformed into a motor response. In this section we will review how communication between brain areas is affected by brain states. This will enable a better understanding of how sensory information is processed in conscious and nonconscious states.

Studies performed in human subjects have long shown that brain regions engage in specific forms of correlated (EEG, fMRI) activity upon the (non-)performance of distinct tasks (Cole et al., 2014; Park and Friston, 2013). This is often investigated in terms of functional connectivity, i.e., the correlation between the activity of different neurons or brain regions. One of the most widely investigated functional networks is the default mode network (DMN). The DMN state arises during quiet wakefulness (a potential micro-state according to our definition) when no task is being performed (Greicius et al., 2003; Lu et al., 2012; Raichle, 2015). Intriguingly, functional coupling between areas which form the DMN drops in the transition from wakefulness to NREM sleep or anesthesia, in both animals and humans (Horovitz et al., 2009; Larson-Prior et al., 2011; Bettinardi et al., 2015). The DMN is usually investigated via fMRI measurements (Daselaar et al., 2010; Larson-Prior et al., 2011; Raichle, 2015) which fail to capture the temporal resolution typical of neural dynamics. Nonetheless, studies performed using electrophysiological approaches have generally confirmed the view that neural networks—especially within the neocortex—become more fragmented when consciousness is lost. Of particular relevance are the studies performed by Massimini and colleagues using a combination of transcranial magnetic stimulation (TMS) and EEG (Massimini et al., 2005; Ferrarelli et al., 2010; Casali et al., 2013; Casarotto et al., 2016). In a series of studies, these authors showed that the spatiotemporal complexity of the TMS-evoked cortical response decreased—with respect to wakefulness in healthy subjects—during NREM sleep (Massimini et al., 2005), non-dissociative anesthesia (Ferrarelli et al., 2010) and disorders of consciousness (Casarotto et al., 2016). This was interpreted as a sign of a decrease in the ability of the cortex to integrate information in nonconscious states (see “The Neuronal Basis of Conscious Sensory Processing” section). Importantly, this functional disconnection is not an all-or-non mechanism, but is rather a graded phenomenon which correlates with the level of residual consciousness (Casarotto et al., 2016). Along the same line, studies performed with techniques enabling higher spatial resolution, such as LFP—recordings (Lewis et al., 2012; Ishizawa et al., 2016) and voltage sensitive dye imaging (Scott et al., 2014), showed a drop in inter-areal communication when consciousness fades.

In spite of this concordance between multiple studies, several key findings stand out. A first incongruence has to do with the capability of cortical areas to integrate multiple sensory modalities during nonconscious states. While this capability is altered during disorders of consciousness in humans (Bonhomme et al., 2012) and in primates (Ishizawa et al., 2016), rodent and ferret studies have consistently shown that cross-modal interactions persist in the neocortex during deep anesthesia (Wallace et al., 2004; Olcese et al., 2013; Foxworthy et al., 2013a,b). Second, studies quantifying state-dependent functional changes in anatomically identified inter-areal connections have shown contrasting results. While connections from the anterior cingulate cortex to the primary visual cortex (V1) in mice are preserved during anesthesia (Zhang et al., 2014), communication from the retrosplenial cortex to V1 loses its functionality (Makino and Komiyama, 2015). These results were obtained by either optogenetically activating feedback-projecting neurons in different brain states and verifying their effect on target regions (Zhang et al., 2014), or by imaging synaptic boutons impinging onto V1 (Makino and Komiyama, 2015). Both types of apparent contradiction could be attributed to the fact that distinct areas as well as pathways are differently modulated by brain state. More recent studies showed that another factor to be taken into account when investigating how brain state modulates neuronal communication is cell-type specificity (Peyrache et al., 2012; Olcese et al., 2016). Indeed, while intra-areal coupling is preserved during NREM sleep, functional connectivity between excitatory but not inhibitory neurons (identified by discriminating action potential waveforms typical of either pyramidal neurons or fast-spiking interneurons) generally drops in NREM sleep compared to wakefulness (Olcese et al., 2016; Storm et al., 2017).

To refine this picture somewhat further, distinct patterns of effective (i.e., directional) connectivity exist at different temporal scales based not only on brain areas, but also on the functional specialization of individual neurons in the context of task performance (Olcese et al., 2018). Specifically, we showed that inter-areal information flow (quantified by Transfer Entropy, Schreiber, 2000) is higher during NREM sleep than during wakefulness at short time scales (i.e., at time scales between 2 ms and 10 ms), between cortical and hippocampal neurons whose activity was modulated during a task rats performed in the active awake micro-state. In this task (during which the activity of about 50% of the recorded neurons was modulated), animals had to choose on which side of a figure-8 maze a reward was to be found, based on visual cue discrimination. Such enhanced information flow was co-occurring with hippocampal sharp-wave ripples, and is consistent with a coordinated reactivation of memory traces that has been shown to occur across hippocampus, neocortex and subcortical structures (Hoffman and McNaughton, 2002; Euston et al., 2007; Ji and Wilson, 2007; Lansink et al., 2009). Conversely, at long time scales (i.e., at time scales between 600 ms and 900 ms), inter-areal information flow was enhanced between neocortical areas and only between neurons whose activity was not modulated by the aforementioned task, without relationship with hippocampal ripples.

Overall, these studies show a complex picture of how brain states modulate communication between brain areas. A net drop in both functional connectivity and information flow between cortical areas is consistently observed when consciousness fades, yet neuronal subpopulations may deviate from this pattern as a consequence of the interplay between multiple factors: anatomical location, cell type, temporal scale and functional specialization. As an example, the reduced functional coupling between cortical areas that we reported to occur in NREM sleep in (Olcese et al., 2016) is mirrored by a specific increase in information flow between functionally-defined neuronal populations (Olcese et al., 2018). These forms of residual communication possibly serve diverse functions such as sleep-dependent memory consolidation. Nevertheless, they are unable to sustain conscious sensory processing, and are therefore highly informative about which forms of communication are instead necessary. Specifically, inter-areal communication (in terms of both correlations and information flow) must be preserved not only for specific neuronal subpopulations or temporal scales (which during sleep may play a role in functions including information reprocessing and memory consolidation), but rather across all circuital elements, in order to allow conscious processing.

All studies we mentioned so far focus on comparing neural connectivity across behavioral macro-states, but how do micro-states affect inter-areal communication? Within wakefulness, task-engagement, attentional mechanisms and stimulus awareness have all been shown to modulate sensory-evoked responses via a strengthening of top down projections from higher order areas to sensory cortices (Lamme and Roelfsema, 2000). The functional circuitry underlying this phenomenon has been especially investigated in the context of whisker-mediated tactile perception in mice. Specifically, it was shown that projections from secondary onto primary somatosensory cortex induce a delayed bout of depolarizations which follows direct sensory-evoked responses (Sachidhanandam et al., 2013; Kwon et al., 2016) and correlates with perception (see also “The Neuronal Basis of Conscious Sensory Processing” section). Similarly, a top-down projection from the secondary motor cortex onto layer 5 pyramidal neurons of the primary somatosensory cortex enhances perceptual accuracy for whisker-related stimuli (Manita et al., 2015), a mechanism which seems to be mediated by dendritic computations (Takahashi et al., 2016). This small selection of studies indicates that, while inter-areal communication is generally necessary for conscious processing—as we discussed in the previous paragraphs—some pathways (and specifically feedback projections) are especially relevant to this mechanism.

What can be concluded from this overview of how brain state influences communication between neurons? The view that a drop in cortical inter-areal communication is a hallmark of nonconscious states can be considered a bit simplistic, as key forms for inter-areal communication persist and can even be enhanced during SWS (Olcese et al., 2016, 2018). First, slow oscillatory patterns are strongly correlated between areas (Volgushev et al., 2011) and have been shown to be traveling waves (Massimini et al., 2004). Strikingly, these waves predominantly move from frontal to posterior regions, i.e., along the frontoparietal, top-down pathway that has been proposed to be essential for conscious processing (Sergent and Dehaene, 2004)—but see (Pennartz, 2015, 2018; Boly et al., 2017). Second, even precise patterns of spiking activity are maintained and transferred between areas during SWS sleep, as shown by many studies on the reactivation of neuronal traces during sleep (Hoffman and McNaughton, 2002; Euston et al., 2007; Ji and Wilson, 2007; Lansink et al., 2008, 2009; Bermudez Contreras et al., 2013; Pennartz, 2015). In addition to the mere involvement of specific anatomical pathways in inter-areal communication, what seems to be essential for discriminating conscious and nonconscious brain states is a particular type of neuronal dynamics that can be instated across the thalamocortical system. In a fully synchronized state such as SWS, mainly one simple type of neuronal dynamics (i.e., alternations between UP and DOWN micro-states) is at play across areas (Massimini et al., 2004). Conversely, a generalized, broad-band desynchronization during the awake state (especially prominent during active wakefulness) does not allow to reliably transfer sensory information, and attentional mechanisms are required to enhance synchrony—and thus signal transmission—between subsets of brain regions and at specific (gamma, beta) frequency bands (Bosman et al., 2012; van Kerkoerle et al., 2014; Bastos et al., 2015; Fries, 2015). An intermediate degree of synchrony in which some specific inter-areal connections—such as feedback projections impinging onto sensory cortices (Manita et al., 2015)—are enhanced, will allow specific cell ensembles to communicate, but prevents an overly massive, nonspecific synchrony from arising (“sparse synchrony;” Pennartz, 2015). The question remains, however, of whether such forms of communications are only a correlate of conscious brain states, or rather constitute the neuronal substrate of perception.

In the next section we will discuss how differences between neuronal activity (in terms of single neurons, ensembles and inter-areal communication) between conscious and nonconscious brain states can provide key elements to better understand conscious sensory processing, in the context of neurally grounded theories of consciousness.

## The Neuronal Basis of Conscious Sensory Processing

As already noted, sensory neural systems continue to respond to stimuli in the absence of consciousness, for instance during anesthesia or deep (NREM) sleep, but also in low-arousal micro-states during wakefulness. How can this be? Apparently, simply generating a sensory response is not sufficient to result in a conscious experience, not even at the level of the neocortex or thalamus, which are the two structures most heavily implicated in conscious sensing (Koch, 2004). The disconnection between sensory responses and conscious experience can be grasped from a theoretical viewpoint: if all that a sensory input gives rise to is activation of a single type of feature detector (e.g., neurons responding to one orientation of a visual grating), then the neural system has no way to deduce, or represent, all visual or other modal properties of the scene or situation. Indeed, a single type of feature detector can only report on orientation, but not on color, spatial frequency, spatial position in the visual field, etc. Furthermore, a single-detector system is incapable of identifying the signal as pertaining to—for instance—orientation, because the system lacks information as to what a transmitted numerical value is about. It only represents or “knows” that a certain level of spiking activity is present. This argument can be extended to a situation where a variety of feature detectors is activated: if their activity is not integrated or combined (i.e., if they are disconnected), the neural system cannot construct an experience with modally distinct properties (Pennartz, 2009).

Various neuroscience-related theories have dealt with the relationship between neural activity and consciousness in different ways. Here we will briefly discuss only two of these theories and compare them with inferential accounts of consciousness. First, the GNW theory mainly focusses on how sensory stimuli gain access to neural systems broadcasting the information across a wide range of brain structures, so that this information quickly reaches systems for motor responding, storage in working memory, or other cognitive functions such as attention (Dehaene and Changeux, 2011). This theory is therefore mainly concerned with information-distributing aspects of conscious processing and postulates that its neural substrates are located primarily in fronto-parietal systems. It is less concerned with phenomenology, i.e., the properties characteristic of subjective experience (see “Introduction” section). However, GNW theory does postulate that conscious processing of a sensory stimulus depends on the stimulus information passing a threshold for “ignition,” i.e., the signal must be strong enough to unleash an explosive activity resulting in the broadcasting of information throughout the neuronal workspace. Ignition may result either as a consequence of feedforward mechanisms (i.e., bottom-up transfer of high stimulus salience), or as a consequence of top-down, attentional resources being directed towards a given stimulus—see e.g., (Buschman and Miller, 2007; Gaillard et al., 2009; van Vugt et al., 2018). This ignited activity would be particularly manifested in frontoparietal systems. Much discussion revolves around the question of whether frontal activity correlates more strongly with generating motor responses to sensory stimuli than with conscious sensing *per se*, which (in the visual domain) has been correlated to neural activity in posterior, higher visual areas (Pennartz, 2015; Koch et al., 2016).

The IIT (Tononi et al., 2016) postulates that sensory systems in the brain are required to engage in causally effective interactions with each other and with other areas in order to give rise to conscious experience. Such causal interactions between neurons are characterized by differentiation as well as integration within the system: the pattern of neural connections must be sufficiently varied or differentiated to guarantee specific content, and must be simultaneously integrated to account for the subject having only one single, unified experience. IIT has a particular focus on explaining differences between conscious/nonconscious brain states in terms of high vs. low levels of integration, while leaving the question of when a sensory stimulus gives rise to perception (both in terms of whether it is perceived and what is perceived) more open. The concept of integration is a general one in neuroscientific terms, as it does not specify the underlying circuit-level mechanisms. Integration of information is considered here in juxtaposition to the view that conscious experience is particularly manifest by subjects sustaining lively, rich experiences and exhibiting complex, planned behaviors typically associated with conscious representations—and which necessarily require the dynamic interaction between neuronal circuits across the whole brain hierarchy.

Therefore, we feel it is timely to also pay attention to a third, neurally grounded theory of consciousness—the inferential or predictive coding (PC) account (Hobson and Friston, 2014; Pennartz, 2015), which has a particular relevance for sensory processing and attempts to characterize the circuit-level neural substrate of perception.

### The Predictive Coding Account of Conscious Sensory Processing

PC has been developed into a growing computational framework, and pays tribute to the Kantian and Helmholtzian notion that what we perceive is not the constellation of exact physical features of our environment, but rather the inferred causes of the sensory inputs our brain receives (Gregory, 1980; Lee and Mumford, 2003; see Wolpert et al., 1995). This is in line with the well-known fact that our brains are fed with sensory input exclusively via the cranial nerves and spinal cord ascending pathways, which all use the same type of signal (action potential) to convey information to the brain. Locally, e.g., within the visual cortex, no intrinsic knowledge is available to deduce that sensory inputs are of a visual nature (Boring, 1950; Block, 2005; Pennartz, 2009). In other words—nothing differentiates individual action potentials generated as a consequence of, for instance, an auditory or a visual stimulus. Amongst other theories of consciousness, such as GNW and IIT, the PC framework stands out for its aim to account for this interpretational (or inferential) aspect of conscious experience. Because of this focus, it is in a relatively favorable position to address perceptual phenomena such as illusions, positing that our brain generates a “best guess” representation of what is going on in the world around us, which leaves open the possibility of false or inaccurate inferences. Here, we will first provide a short overview of the PC framework, and will then discuss its relevance for the study of conscious sensory processing.

Neural network versions of PC and related accounts were developed by e.g., Dayan et al. (1995), Rao and Ballard (1999) and Spratling (2011). For instance, Rao and Ballard (1999) designed a two-layer neural network in which sensory inputs were relayed by an input layer to a higher layer that generated a predictive representation of the actual sensory input. This predictive representation offers an inference as to what causes the sensory input. Importantly, “predictive” does not necessarily mean predictive-in-time; the prediction can be as much about inferring the causes of current sensory input as it may pertain to future inputs or states. The predictive representation is fed back to the input layer via recurrent projections. Next, local circuits in or near the input layer compute the discrepancy between the input and the prediction, and this error (or mismatch) signal is then used to train the network to generate more accurate predictions about the input in the future. This unsupervised updating of the internal model continues until the error signal is minimized. Rao and Ballard (1999) showed that even a two-layer PC network can explain extra-classical, receptive field properties of visual cortical neurons.

Friston (2010) incorporated PC in his formulation of a Free-Energy principle for optimization of action, perception and learning. He also studied the role of motor actions in generating predictions and acquiring sensory feedback to guide predictive learning (“active inference”). Seth (2013) applied the PC framework to study the genesis of models of internal body states in relation to emotions and subjective feelings. Bastos et al. (2012) proposed a theoretical scheme of how errors and predictive representations may be transmitted via feedforward and recurrent pathways in the visual cortical system, linking this transmission to gamma and beta activity in LFPs of superficial vs. deep cortical layers, respectively. Recently, Dora et al. (2018) presented a model for PC in deep, multi-layer networks, showing that the model—trained by unsupervised learning—can generate effective inferred representations of sensory inputs (images), even when the network had not been exposed to these inputs before. When the network was put to operate in the generative mode—meaning that sensory inputs were absent and inferred representations were triggered by activation of higher layers, thus crudely simulating imagery—it was shown to reproduce the original inputs that gave rise to these inferred representations after training. Thus, this model illustrates aspects of perceptual inference as well as imagery, understood as an internally driven mode of active neural representation.

How exactly may the PC framework contribute to the development of theories of consciousness? Above we already hinted at its basic capabilities in explaining inference and imagery, but it should be noted that certain kinds of inference may occur non-consciously. For instance, we perform many actions in an automated, nonconscious fashion while using inferred information (e.g., automatically grabbing a coin from a collection with various sizes). A foremost question to ask when comparing PC to properties of conscious experience is: which component—if anything—of a PC model would correspond to an experienced representation? The actual sensory input cannot correspond to this, because this is not what is being perceived, but rather what the brain makes inferences about. The feedforward, ascending signals present the discrepancies between actual inputs and inferred causes. However, it is not an error that we perceive, but rather the inferred representation generated by one or more higher layers of the system. It is this joint (multi-layer) predictive representation that holds several properties that we can roughly associate with conscious experience: (i) interpretation or inference, and thus the property of being prone to illusions; and (ii) a capacity to switch or alternate between perception (externally driven) and imagery (internally driven). How other attributes of conscious experience (see “Introduction” section) may be explained by PC remains largely to be explored, but it is of note that the concept of “integration” is inherent to the construction of predictive representations, dependent as this process is on layer-to-layer interactions (for a discussion of multimodal richness, immersiveness, situatedness and first-person perspective, see Pennartz, 2015).

Despite the increasing prominence PC theory has gained in relation to consciousness and its neural substrates, the question of whether inference can be accomplished nonconsciously (and how this relates to macro- and micro-states) deserves further scrutiny. First, we argue that inference plays a central role in many processes contributing to perception. For instance, gauging the color constancy of an object seen under different spectral conditions (such as bright daylight vs. sunset) is, in a broad sense, a process of inference. Likewise, distinguishing objects against their background (figure-ground segregation) and the grouping of features into a single object are functions implying inference (various neural systems with a different history of learning may group features or segment objects differently). Thus, inference is not limited to illusions, and is involved in many of the bread-and-butter operations underlying perception.

Second, the evidence for selectively coupling different forms of inference to consciousness varies per study and form being studied. The Kanizsa triangle illusion, for instance, depends on the presence of inducers—the three aligned Pacman figures and the three pairs of lines that seem to underlie the illusory white triangle we perceive as being superimposed upon these inducer stimuli. Harris et al. (2011) used continuous flash suppression to render inducers of this illusion invisible. Subjects responded randomly when asked to report whether the triangle was pointing to the left or right, suggesting that the underlying inferential process is associated with consciousness. Similarly, inference on color constancy appears to be linked to consciousness, because it is absent in the lesioned hemi-field of blindsight patients (Barbur and Spang, 2008). Figure-ground segregation and grouping based on low-level Gestalt features are also thought to depend on consciousness, as they are largely absent under anesthetized conditions or during perceptual masking. However, overall the results depend on the precise task conditions and perceptual abilities of subjects (Marcel, 1998), making it difficult to assess which types of inference are firmly connected to consciousness and which are not.

**Figure 4:**
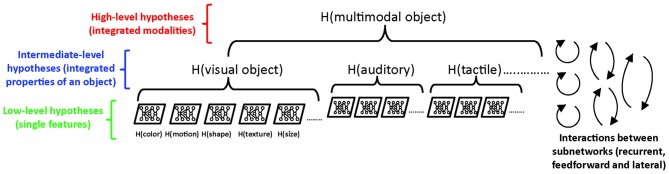
The inferential account of conscious sensory processing. Illustration of the hypothesis that conscious sensory processing is organized according to low, intermediate and high representational levels (see Pennartz, 2015). In this scheme, representations correspond to predictions or hypotheses (rendered here as H(…)). A low-level prediction or hypothesis (green) pertains to a singular feature within a sensory modality, pertaining to an object or location in the environment (e.g., H(color) is the hypothesis that a visual object is of a certain color). The intermediate level (blue) is exemplified by H(visual object), the hypothesis that a visual object has several properties integrated across the low-level predictions. The highest level of representation (red) integrates across several sensory modalities (vision, audition, somatosensory and (not shown) olfaction, taste, sense of balance) and is rendered here as the hypothesis on a multimodal object. For instance, a grabbing of a piece of paper in one’s hand generates a joint inference on its visual, tactile and auditory properties. Already at the lowest level, predictions are learned and generated within a feedforward and recurrent multi-layer architecture which may well stretch across several connected cortical areas (e.g., V1, V2, V3 and V5 for visual motion). Note that motor or situational aspects, including the object’s position in space, are not taken into account in this scheme. In addition, a prediction on one particular feature × may well depend on predictions on other features y and z (etc.), for instance when assessing object shape from colored patches, texture and disparity cues. Thus, hypotheses are can be conditional on each other, which may be implemented by interactions between sub-networks. Horizontal dotted lines denote that the potential list of relevant features or sensory modalities can be extended. Curved arrows indicate the various types of interaction between sub-networks (recurrent, feedforward and lateral).

Thus, a central problem to study is how the various types of inference—whether made consciously or not—are integrated into an overall experience, and where and how this is accomplished in the brain. As argued more extensively elsewhere (Pennartz, 2015), it would be erroneous to seek the overall “interpretation” of all current sensory inputs in a higher brain area (e.g., prefrontal cortex) that receives convergent inputs from lower-level (sensory) cortical areas. All that higher-area neurons receive from lower areas are synaptic inputs, arising from trains of action potentials, which hold no explanatory power for phenomenology when considered by themselves. Instead, we argue that an overall perceptual interpretation arises at a higher representational level of the system, where “level” means a conceptual level of organization and representation, analogous to Marr’s distinction between implementational, algorithmic and computational levels (Marr, 1982). Translated to the PC framework, this means it is productive to make a distinction between low-level predictive systems (such as the two-layer or multi-layer architectures noted above; Rao and Ballard, 1999; Dora et al., 2018) that are only concerned with e.g., color or motion vision, vs. high-level, integrated predictive systems. Many low-level predictions can then be subsumed under a high-level predictive system, which serves to integrate the low-level representations and unify them into a perspectival, multimodal experience (Figure 4).

Considering the anatomical correlates of low vs. high-level predictions, low-level predictions in the visual modality concern individual submodalities such as color, motion, texture, shape and size, and of these, for instance, motion can be associated with area V5/MT (Zihl et al., 1983; Zeki, 2001). High-level predictions pertain to the level of single modalities (e.g., vision, including all of its submodalities) or, ultimately, to the highest level of multimodal representations that we can associate with phenomenology. Such high-level predictions play out at the level of large, integrated anatomical aggregates comprising both sensory cortices and higher, associational areas such as the parietal cortex (Pennartz, 2018). Hence, the experimental evidence underscoring the importance of long-range cortical communication for consciousness is just as compatible with the PC account as it is with IIT or GNW.

In conclusion, what distinguishes conscious and nonconscious sensory processing in this proposal is not that inference is an exclusive privilege of conscious systems, but that all inferential processes together give rise to a higher representational level of “super-inference” in which they are integrated and unified. Note that the aspects of integration and multimodal richness are taken on board in this account, and that especially the multi-level aspect of this representational theory needs further elaboration through computational modeling and experiment.

### Inferential Accounts: Comparison to Experimental Data

Originally, we asked how it is that sensory systems in the cortex continue to respond to stimuli in the absence of consciousness, for instance during anesthesia or NREM sleep, but also during wakefulness when a stimulus is not perceived. Based on the brief exposé that we gave on PC, it can now be hypothesized that responses to stimuli, at least in the primary sensory cortices, may reflect either a bottom-up sensory input or a low-level inference which fails to become integrated into a high-level predictive representation. At the level of the primary visual cortex V1, for instance, responses to visual stimuli reflect inputs from the retina-LGN (lateral geniculate nucleus of the thalamus) subsystem, which continue to be transmitted under anesthesia or NREM conditions. It has also been noted that top-down (or recurrent) processing is lacking or diminished under anesthesia (Lamme et al., 1998), which fits in this account because it may indicate that either predictive representations in higher visual areas are not properly formed, or are formed but fail to integrate with those in lower areas such as V1. It should be noted that the relative intactness of primary sensory responses under nonconscious conditions is also broadly compatible with GNW and IIT. For GNW, this holds because the “ignition threshold” may be set at higher anatomical stages of cortical processing than the primary sensory cortices. For IIT, it is not the presence of a sensory response as such that determines whether the subject becomes conscious of it, but rather whether it is processed in a sufficiently integrated manner, yielding sufficient causally effective communication and differentiation.

Another line of testing inferential accounts is to study how sensory prediction error signals may be generated in the brain, and whether they persist under nonconscious conditions. In a behavioral paradigm where a virtual reality setup was used to dissociate locomotor movement of mice from optic flow (which would normally be predictable from locomotion), Keller et al. (2012) found indications for sensorimotor mismatch coding in superficial layers of mouse visual cortex. In a recent follow-up study, Leinweber et al. (2017) studied the projection from frontal cortical areas A24b and M2 (secondary motor cortex) to visual cortex as an anatomical substrate that may potentially convey predictive motor information to the visual system. In a task variant where the optic flow resulting from turning movements was left-right inverted, they found that correlations between neural activity and behavioral responses adapted to diminish mismatches to visual flow, in line with a PC account. Note how this relevance of feedback connections is in line with the role that we described for connections between motor and tactile areas in the context of whisker perception (“How Communication Between Brain Areas Varies Across Brain States, and How This Affects Sensory Processing” section).

Mismatch signals have also been investigated in human EEG/MEG responses, where the mismatch negativity (MMN) signal arises from detecting deviations from a regular, temporally repetitive series of stimuli. A recent study (Strauss et al., 2015) used an auditory paradigm that included both short-term (local) and long-term (global) regularities, according to a hierarchical order. They found that, while the local (low-level) mismatch response remained present during all sleep stages, sleep abolished the global mismatch response (reflected in the P300 component). This would align well with the current hypothesis that inferences can be generated at multiple representational levels, and that particularly low-level inferences can be produced nonconsciously, whereas high-level inferences correspond to conscious experience. It must be noted though that the notion of “level” used here may be different from that implied by the paradigm used in Strauss et al. (2015), and that time series predictions constitute a special case in the family of PC paradigms.

The experimental findings reviewed in section “Sensory-Evoked Neuronal Responses Across Brain States” emphasize a prominent role in conscious representations for sparsity of neuronal activity. This means that having a sufficient population sparsity in the coding of sensory stimuli—which is promoted in a desynchronized network micro-state—is instrumental in the generation of conscious inferences. This role can be interpreted in the context of representational or inferential accounts, because a population state of high sparsity corresponds best with the coding of specific features pertaining to the presented stimulus, whereas neural coding of other (non-actual) features should be preferably absent. The ensuing micro-state of “sparse synchrony” is deemed suitable to convey such highly specific sensory information to neural ensembles in other cortical areas (Pennartz, 2015). Although the notion of sparse synchrony seems to be compatible with IIT in the sense that it corresponds to having differentiation in the neural population, IIT is less outspoken about how much sparsity would be preferred, as it more strongly emphasizes the importance of causally effective transfer of information throughout a network. On the other hand, GNW gives more emphasis to the magnitude of the neural activity induced by sensory signals (as a consequence of stimulus intensity and/or focused attentional resources) relative to the hypothesized “ignition threshold” and it remains to be investigated how “magnitude” should be defined here relative to sparse population coding. Rather than massive sensory activation, surpassing a perceptual threshold, the data appear to be more compatible with a “sparse synchrony” threshold that has to be met in several connected sensory cortical areas to give rise to conscious sensing.

Returning to a set of findings on communication between cortical brain areas (“How Communication Between Brain Areas Varies Across Brain States, and How This Affects Sensory Processing” section), we would argue that the global decrease in long-range communication, found for instance during NREM sleep and anesthesia, is compatible with inferential accounts of consciousness as well as with GNW and IIT. The spatiotemporal complexity of TMS-evoked cortical responses decreases—with respect to wakefulness in healthy subjects—during NREM sleep (Massimini et al., 2005), anesthesia (Ferrarelli et al., 2010) and disorders of consciousness (Casarotto et al., 2016) has been interpreted as a decrease in the ability of the cortex to integrate information in nonconscious states. IIT appears therefore to be in line with these experimental findings (Tononi et al., 2016). These results on complexity can be partially explained by the observation that propagation and reverberation of TMS evoked cortical activity is prevented by DOWN states arising during SWS or other nonconscious conditions (e.g., coma). While this explanation may seem simple vis a vis the complexity of conscious processing, it is plausible and effective at a mechanistic, electrophysiological level.

As regards the inferential accounts, it is noted that high-level predictive representations (under which many “simpler” low-level representations are subsumed) can only be formed given sufficient communication between brain areas, which are anatomically located in lower and higher parts of a sensory (and multisensory) hierarchy. This renders the account well compatible with the findings on long-range communication. The importance of long-range communication has also been stressed by GNW theory, because it is this type of information that is deemed important here for globally broadcasting information we are conscious of. Thus, correlations between changes in conscious (micro-)state and in long-range communication are considered highly relevant yet do not strongly discriminate between the three theories under consideration here. An exception may be the finding that, during NREM sleep, some pairs of brain areas show enhanced rather than diminished directed interactions (e.g., between specific subpopulations of neurons located in different cortical areas, Olcese et al., 2018). Such enhancements in specific cortical circuits normally considered essential in conscious processing may be difficult to explain within the IIT framework. The inferential account places emphasis on constructing representations in sensory cortical systems. Therefore, enhanced interactions between cortical areas not leading to conscious representations (for instance involved in distinct functions associated with sleep, such as memory processing, Qin et al., 1997; Pennartz et al., 2002; Sirota et al., 2003) may well be present during nonconscious states and do not present a challenge for the inferential account.

## Discussion and Conclusion

Here, we have presented a review of the different ways in which sensory stimuli are processed by cortical areas across conscious and nonconscious brain states. First, we have argued that the classical definition of brain states (e.g., wakefulness and the various sleep stages) needs to be updated to take into account that such macroscopic states are highly heterogeneous, and that they can be subdivided in distinct micro-states. Such micro-states can be distinguished based on their different patterns of neural activity, but more importantly, based on how sensory stimuli are processed. Next, we have examined how sensory processing varies across macro- and micro-brain states, at the level of single neurons, ensembles, and inter-areal communication. While sensory-evoked responses are largely preserved across conscious and nonconscious brain states, differences emerge when focusing on natural (e.g., complex multisensory scenes) rather than simple sensory stimuli, and when considering the fine structure of spiking and inter-areal communication patterns. In particular, we can conclude that what characterizes brain states capable of sustaining conscious sensory processing is the presence of an intermediate level of desynchronization. We refer to this condition as “sparse synchrony.” This corresponds to a micro-state in which the strong level of synchrony present in nonconscious states such as NREM sleep and anesthesia (e.g., cortical slow wave activity) is lost, but specific forms of integrated activity (e.g., feedback projections from higher order to primary cortices) remain possible. How is it that this sparse synchrony enables perception? That an intermediate level of integration is key to enable consciousness has been highlighted by different neural-based theories of consciousness such as IIT and GNW. Here we focus on the PC framework, which is uniquely focused on sensory processing. As we showed, the PC (inferential) framework provides a valid platform to explain how different forms of neural dynamics that characterize the distinct brain states affect sensory processing, how percepts may be explained as “best guess” representations and should be considered as a powerful theoretical framework alongside other theories of consciousness.

## Author Contributions

UO, MOL and CP wrote the manuscript.

## Conflict of Interest Statement

The authors declare that the research was conducted in the absence of any commercial or financial relationships that could be construed as a potential conflict of interest. The reviewer, AP declared a past co-authorship with one of the authors, UO to the handling Editor.
